# Identification of the Protein Kinases Pyk3 and Phg2 as Regulators of the STATc-Mediated Response to Hyperosmolarity

**DOI:** 10.1371/journal.pone.0090025

**Published:** 2014-02-25

**Authors:** Linh Hai Vu, Tsuyoshi Araki, Jianbo Na, Christoph S. Clemen, Jeffrey G. Williams, Ludwig Eichinger

**Affiliations:** 1 Center for Biochemistry, Institute of Biochemistry I, Medical Faculty, University of Cologne, Cologne, Germany; 2 College of Life Sciences, Wellcome Trust Building, University of Dundee, Dundee, United Kingdom; Cardiff University, United Kingdom

## Abstract

Cellular adaptation to changes in environmental osmolarity is crucial for cell survival. In *Dictyostelium,* STATc is a key regulator of the transcriptional response to hyperosmotic stress. Its phosphorylation and consequent activation is controlled by two signaling branches, one cGMP- and the other Ca^2+^-dependent, of which many signaling components have yet to be identified. The STATc stress signalling pathway feeds back on itself by upregulating the expression of STATc and STATc-regulated genes. Based on microarray studies we chose two tyrosine-kinase like proteins, Pyk3 and Phg2, as possible modulators of STATc phosphorylation and generated single and double knock-out mutants to them. Transcriptional regulation of STATc and STATc dependent genes was disturbed in pyk3^−^, phg2^−^, and pyk3^−^/phg2^−^ cells. The absence of Pyk3 and/or Phg2 resulted in diminished or completely abolished increased transcription of STATc dependent genes in response to sorbitol, 8-Br-cGMP and the Ca^2+^ liberator BHQ. Also, phospho-STATc levels were significantly reduced in pyk3^−^ and phg2^−^ cells and even further decreased in pyk3^−^/phg2^−^ cells. The reduced phosphorylation was mirrored by a significant delay in nuclear translocation of GFP-STATc. The protein tyrosine phosphatase 3 (PTP3), which dephosphorylates and inhibits STATc, is inhibited by stress-induced phosphorylation on S448 and S747. Use of phosphoserine specific antibodies showed that Phg2 but not Pyk3 is involved in the phosphorylation of PTP3 on S747. In pull-down assays Phg2 and PTP3 interact directly, suggesting that Phg2 phosphorylates PTP3 on S747 *in vivo*. Phosphorylation of S448 was unchanged in phg2^−^ cells. We show that Phg2 and an, as yet unknown, S448 protein kinase are responsible for PTP3 phosphorylation and hence its inhibition, and that Pyk3 is involved in the regulation of STATc by either directly or indirectly activating it. Our results add further complexities to the regulation of STATc, which presumably ensure its optimal activation in response to different environmental cues.

## Introduction

Virtually all cells, even individual cells in multi-cellular organisms, are subjected to changes in the osmotic environment that are sometimes extremely rapid. In order to survive cells have to sense these changes and elicit an appropriate response that allows them to adapt. When *Dictyostelium discoideum* cells are faced with a hypertonic environment a complex response is triggered. It starts with signal sensing and transduction and leads to changes in cell shape, the cytoskeleton, transport processes, metabolism and gene expression [1], [2]. STATc (Signal Transducer and Activator of Transcription c), one of the four STAT proteins encoded by *D. discoideum,* is an important mediator of the transcriptional response to hyperosmolarity as it regulates 20% of the induced genes [2], [3].

In mammals, STAT1 and STAT3 are normally activated by cytokines but can also be activated by hyperosmotic stress [4]. Mammalian STATs are generally activated by JAK (Janus kinase) family members through tyrosine phosphorylation [5]. In contrast, *D. discoideum* does not encode a JAK ortholog. However, the genome encodes a large number of tyrosine-kinase like (TKL) proteins which constitute STATc kinase candidates [6]. Hyperosmotic shock is suggested to activate two parallel, oppositely acting pathways, which are under control of the second messengers cGMP and Ca^2+^, respectively, and ultimately cause phosphorylation of STATc on Tyr922 [7]. Phosphorylated STATc dimerises, translocates to the nucleus, and controls gene expression. Within the former pathway, STATc can be activated by the membrane-permeable cGMP analogue 8-Br-cGMP, which acts on the cGMP-binding protein C (GbpC), a founding member of the ROCO family of protein kinases that also contain LRRK2, the protein most frequently mutated in familial Parkinson’s disease [3], [8]. The activation of STATc by 8-Br-cGMP but not by osmotic stress is lost in a gbpC^−^ strain. Furthermore, hyperosmotic stress-induced tyrosine phosphorylation of STATc was still observed in a *D. discoideum* mutant, wherein both known guanylate cyclases (GCA and sGC) were disrupted [3]. This can be explained by the parallel activation of the Ca^2+^ branch in response to hyperosmolarity [7]. Recently it was shown that the TKL protein Pyk2 directly phosphorylates STATc on Tyr922 in response to the chlorinated hexaphenone DIF-1, which activates STATc during development [7], [9]–[11].

Another essential player in the complex regulation of STATc is the constitutively active protein tyrosine phosphatase 3 (PTP3) [7], [12]. PTP3 is localised in the cytosol of unstimulated cells and accumulated at endosomes, when cells were subjected to hyperosmotic stress [13]. Overexpression of PTP3 inhibited STATc tyrosine phosphorylation, whereas overexpression of a dominant inhibitor of PTP3 led to constitutive STATc phosphorylation and nuclear localisation [12]. An ortholog of the mammalian E3 ubiquitin ligase Cbl, CblA, was found to act as a positive regulator of STATc phosphorylation by down-regulating PTP3 [14]. Exposure to hyperosmotic stress or to agents that elevated intracellular Ca^2+^ levels induced phosphorylation of S448 and S747 of PTP3 and inhibited its enzymatic activity [7].

Here we characterise two further modulators of the STATc signalling cascade, the TKL proteins Pyk3 and Phg2, which were selected based on previous microarray results and BLAST searches [2]. We analysed single knock-out mutants for *pyk3* and *phg2*, a double knock-out mutant of both genes, as well as GFP-STATc, Myc-PTP3 and Myc-Phg2 overexpressing Ax2 wild-type and mutant cells with respect to transcriptional regulation, STATc and PTP3 phosphorylation, and STATc nuclear translocation. We present evidence that both TKL proteins modulate STATc-signalling and that Phg2 but not Pyk3 acts as a PTP3 inhibitor.

## Materials and Methods

### Strains and Cell Culture

*D. discoideum* Ax2 wild-type and mutant cells (Table 1) were grown at 21°C either on SM agar plates with *Klebsiella aerogenes* as food source [15] or in Ax2-medium (for 1 l: 14.3 g bacteriological peptone, 7.15 g yeast extract, 18 g maltose, 0.62 g Na_2_HPO_4_×2H_2_0, 0.49 g KH_2_PO_4_, pH 6.7), that contained 40 µg/ml dihydrostreptomycinsulfate and in the case of mutant strains in addition 10 µg/ml blasticidin or 10 µg/ml G418, on plates (90 mm diameter) or in Erlenmeyer flasks with shaking at 160 rpm [16]. For cell biological work, cultures were harvested at a density of 3–4×10^6^ cells/ml. For treatment with 200 mM sorbitol, 20 mM 8-Br-cGMP, 30 µM BHQ or 5 µM thapsigargin, cells were washed twice with Soerensen phosphate buffer (2 mM Na_2_HPO_4_, 14.6 mM KH_2_PO_4_, pH 6.0), resuspended in the same buffer at 2×10^7^ cell/ml, and developed under shaking at 200 rpm for 4 hours. Treatment was performed for 5 to 15 minutes depending on the assay.

**Table 1:** *D. discoideum* mutant strains used in this study.

Strains	Summary	Reference
pyk3^−^	Pyk3 null mutant	this study
phg2^−^	Phg2 null mutant	this study
pyk3^−^/phg2^−^	Pyk3/Phg2 double null mutant	this study
STATc^−^	STATc null mutant	[10]
Ax2/[act15]:Myc-PTP3	Overexpression of Myc-PTP3 in Ax2	this study
phg2^−^/[act15]:Myc-PTP3	Overexpression of Myc-PTP3 in a Phg2 null background	this study
pyk3^−^/[act15]:Myc-PTP3	Overexpression of Myc-PTP3 in a Pyk3 null background	Tsuyoshi Araki, unpublished
Ax2/[act15]:Myc-Phg2	Overexpression of Myc-Phg2 in Ax2	this study
Ax2/[act15]:GFP-STATc	Overexpression of GFP-STATc in Ax2	this study
pyk3^−^/[act15]:GFP-STATc	Overexpression of GFP-STATc in a Pyk3 null background	this study
phg2^−^/[act15]:GFP-STATc	Overexpression of GFP-STATc in a Phg2 null background	this study
pyk3^−^/phg2^−^/[act15]:GFP-STATc	Overexpression of GFP-STATc in a Pyk3/Phg2 double null background	this study

### Molecular Biological Methods

All *Dictyostelium* mutant strains were generated in the AX2 background. Gene replacement constructs were generated in the pLBPLP vector where the blasticidin resistance (bsr) cassette is flanked by *loxP* sites [17]. For the phg2^−^ construct, a PCR amplified fragment of 507 bp (5′-homology arm; forward primer: 5′-GCGGGATCCGGATGACCAACAACCATTAC-3′; reverse primer: 5′-GCGCTGCAGACGCCCAAAATTAAGCTTTAG-3′) was cloned into the BamHI and PstI sites and a 670 bp fragment (3′-homology arm; forward primer: 5′-GCGAAGCTTCTAGATGTTGGGATGAGAACCCTGAC-3′; reverse primer: 5′-GCGGTCGACCATTTAGAGTGCTTTTATTACCTTC-3′) into the HindIII and SalI sites of the vector. Upon homologous recombination of the ‘restriction enzyme’ linearized targeting construct, the genomic region of the *phg2* gene encompassing the TKL domain was replaced by the bsr cassette (Fig. S1). Similarly, the pyk3^−^ construct was created. A PCR amplified fragment of 551 bp (5′-homology arm; forward primer: 5′- CGCGGATCCGAAGGTATGGATCCGATATTGGC -3′; reverse primer: 5′- CGCCTGCAGGCAGGTGGTAAATTTGTAATTG-3′) was cloned into the BamHI and PstI sites and a 656 bp fragment (3′-homology arm; forward primer: 5′- CGCAAGCTTCTTTAGGTATGGAACATCTTC-3′; reverse primer: 5′- CGCGTCGACCTAACTATCAACCTCTTCATC-3′) into the HindIII and SalI sites of the vector. Upon homologous recombination, the bsr cassette of the targeting construct replaced 2.37 kbp of the *pyk3* gene thereby disrupting part of the TKL domain (Fig. S1). To generate the pyk3^−^/phg2^−^ double knock-out strain, the pyk3^−^ strain was first transformed with the pDEX-NLS-cre vector to transiently express the *cre* recombinase [18]. After removal of the *loxP* flanked bsr cassette, blasticidin sensitive pyk3^−^ cells were transformed with the phg2^−^ construct. An independent pyk3^−^ knock-out strain was generated by Tsuyoshi Araki (University of Dundee). The two knock-out mutants were exchanged between the two laboratories and compared in some of the real time PCR and STATc phosphorylation experiments. The plasmids for expression of Myc-PTP3 and GFP-STATc in *D. discoideum* cells have been described elsewhere [3], [12]. The plasmids were introduced into Ax2 wild type cells and the different mutants by electroporation. For the Myc-Phg2 construct the sequence encoding aa 207–1159 of Phg2 was cloned into the Myc-PTP3 expression vector by replacing the PTP3 coding sequence and the plasmid was transformed into Ax2 wild-type cells. Transformants were selected in the presence of either 10 µg/ml blasticidin S or G418 and identified by PCR screening of resistant clones followed by visual inspection under a fluorescence microscope and/or immunological detection of the expressed proteins in Western blots. RNA isolation and real time PCR was essentially done as described [19]. The used primers were purchased from Metabion Corp. (Munich, Germany; Table 2).

**Table 2:** Primer pairs used for quantitative Real-Time PCR analysis.

dictyBase ID	GeneName	Sequence
DDB_G0293532	dstc	Forward: 5′-CAATTACTTTGTGGCACTCG-3′Reverse: 5′-CCAAATTTGAGGGTTACTGG-3′
DDB_G0282145	*ptpC*	Forward: 5′-ACCGATATGGGTATTCGTTC-3′Reverse: 5′-TGTTGTGGTGGGAATTTTAG-3′
DDB_G0283385	*pyk1*	Forward: 5′-AAGTTGGCTTTGGATATTGC-3′Reverse: 5′-GCCATGTAGGGAATACAACC-3′
DDB_G0285321	*pyk2*	Forward: 5′-CTGTACACACTACTAGTGGTGG-3′Reverse: 5′-CTACCTCTAGGTCTCATATGAG-3′
DDB_G0289001	*pyk3*	Forward: 5′-TTTTATGGGTGCCTGTATTG-3′Reverse: 5′-TCATCGCTTAAAGTTGTTGC-3′
DDB_G0283699	*phg2*	Forward: 5′-GAGAACCCTGACAAAAGACC-3′Reverse: 5′-GAGGAAGAAGGCACAACAAC-3′
DDB_G0282895	*morn*	Forward: 5′-ATTGGATCACCACCATTACC-3′Reverse: 5′-TCATCACCACTTGAAGCAAC-3′
DDB_G0293416	*abcB1*	Forward: 5′-CAGCTATCAATGTTTCAGGTG-3′Reverse: 5′-CCACGATACCAAACTGGATCC-3′

Oligonucleotide primers were designed on the basis of sequence information and purchased from Metabion Corp. (Munich, Germany).

### Expression and Purification of Recombinant Proteins

For generation of Pyk3- and Phg2-specific polyclonal antibodies (pAbs), cDNA sequences encoding amino acids 1020–1309 of Pyk3 and 656–706 of Phg2 were amplified by PCR and cloned into the *Escherichia coli* expression vector pGEX-6-P-1 (GE Healthcare Corp.). The fusion proteins were expressed in *E. coli* XL1-blue, purified using glutathione agarose beads, released from the beads through cleavage with PreScission protease, and used for the immunization of rabbits (BioGenes GmbH, Berlin, Germany). For pull-down assays amino acids 207–1159 of *Phg2* were expressed, purified, and eluted with glutathione. For *in vitro* PTP3 phosphorylation we used bacterially expressed GST-PTP3ΔCS [12].

### SDS PAGE and Western Blotting

SDS-PAGE and Western blotting were essentially performed as described [20], [21]). Proteins of total cell lysates from 2–4×10^5^ cells were separated by SDS-PAGE and electrophoretically transferred to a nitrocellulose membrane with a wet blot apparatus at 10 V overnight. Phg2 and Pyk3 polyclonal antibodies were used at a 1∶100 and 1∶40,000 dilutions, respectively. GFP was detected with mAb K3-184-2 [22], total STATc with mAb 7H3 and Tyr^922^-phosphorylated STATc with mAb CP22 [10]. Myc-tagged proteins were detected with anti myc mAb 9E10 at a 1∶1,000 dilution. Ser^448^- and Ser^747^-phosphorylated PTP3 were monitored by using specific PTP3 phospho S448 and S747 antibodies [7]. Secondary antibodies used were anti-mouse and anti-rabbit IgG conjugated with peroxidase (POD) (Sigma, Germany) followed by chemiluminescence detection. Images were recorded and analysed using the Fluorchem SP imaging system (Alpha Innotech, USA).

### Fluorescence Microscopy

Fluorescence microscopy studies were carried out using Ax2 wild-type, pyk3^−^, phg2^−^, and pyk3^−^/phg2^−^ cells ectopically expressing GFP-STATc [3]. 1×10^6^ cells were collected, washed twice with Soerensen phosphate buffer, resuspended in 100 µl of the same buffer, and allowed to settle for 15 min on acid-washed glass coverslips (18 mm diameter). The cells were treated with 100 mM sorbitol for 0 to 8 minutes, immediately fixed with pre-chilled (−20°C) methanol in a petri dish, and incubated at −20°C for 10 min. The coverslips were then washed three times with 500 µl PBG (1x PBS, pH 7.4, 0.5% bovine serum albumin, 0.1% fish gelatine buffer), mounted onto glass slides with the cells facing downwards, sealed with gelvatol, and stored in the dark at 4°C overnight. Images of fixed cells were recorded with the inverted fluorescence microscope (LEICA DMI 6000 B) at 488 nm excitation and 500–550 nm emission and processed using the Leica MM AF software.

### Pull-Down Experiments

Myc-PTP3 was expressed in phg2^−^ cells. 1–6×10^7^ cells were treated with 200 mM sorbitol for 15 minutes and lysed in 1 ml mNP40 lysis buffer (50 mM Tris/HCl, pH 8.0, 150 mM NaCl, 1% NP-40, 50 mM NaF, 2 mM EDTA, 2 mM Na-pyrophosphate, complete EDTA-free protease inhibitor cocktail (1 tablet/50 ml; Roche GmbH)) for 10 min on ice. The cell lysate was centrifuged at 7,000× *g* for 10 min, the supernatant was pre-cleared by incubation with Dynabeads Protein G (Life technologies™) for 30 min at 4°C with rotation, followed by centrifugation at 500× *g* for 1 min. The cleared supernatant was incubated for 30 min at 4°C with 2 µg Myc antibody (mAb 9E10) and then for further 60 min with the Dynabeads Protein G. After incubation the Dynabeads were washed four times with EDTA free mNP40-buffer. 5–10 µg bacterially expressed and purified GST-Phg2 were released from glutathione agarose beads through incubation with 20 mM glutathione, and added in pull-down buffer (20 mM Hepes/NaOH, pH 7.4, 10 mM MgCl_2_, 0.02% NP-40, 30 mM NaCl, 1 mM Glycerol-2-phosphate, 1 mM DTT containing complete EDTA-free protease inhibitor cocktail (1 tablet/50 ml) and 0.2 mM ATP) to the Dynabeads followed by incubation for 2 h at 4°C. For reverse pulldown myc-PTP3 was immunopurified from phg2^−^ cells as above and eluted from the Dynabeads by a pH shift (50 mM Glycin, pH 2.8). The eluted Myc-PTP3 was then incubated in pull-down buffer together with 50 µl GST-Phg2 coated glutathione agarose beads. In both protocols the beads were washed 5–10 times with washing buffer (50 mM Tris/HCl, pH 8.0, 150 mM NaCl), eluted in 50 µl 3 x SDS sample buffer, boiled for 3 minutes, and the proteins were separated by SDS-PAGE followed by Western blot analysis using anti Myc, anti Phg2, and anti GST-antibodies.

## Results

### STATc Differentially Regulates Genes Encoding Signalling Proteins

**Figure 1:**
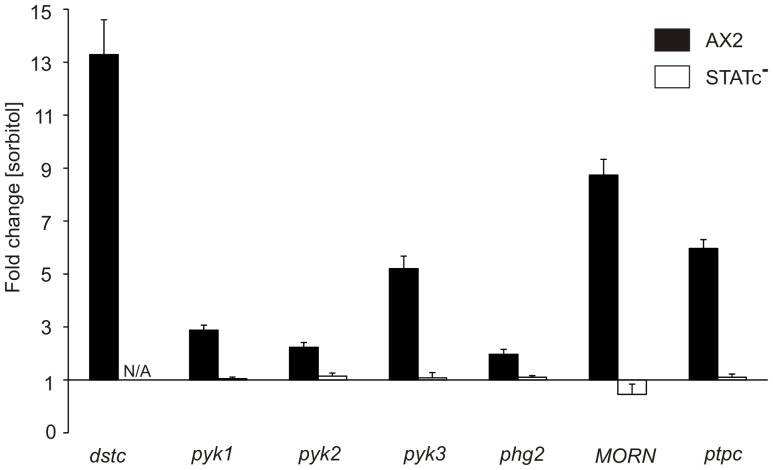
Real time PCR confirms STATc-dependent transcriptional activation of selected genes. The differential expression of seven selected genes (see Table 3) in Ax2 wild-type (black bars) and STATc^−^ (white bars) cells in response to treatment with 200 mM sorbitol for 15 minutes was analysed by quantitative real time PCR. The data are expressed as means of fold change in comparison to untreated cells. Fold changes and standard deviations of six measurements from three independent experiments are shown. N/A: *not applicable.*

STATc is a key regulator of the transcriptional response to hyperosmotic shock and regulates approximately 20% of the differentially expressed genes [2]. We found that a small fraction of the STATc dependent genes was already differentially regulated 15 minutes after subjecting the cells to hyperosmotic conditions. Most interesting of these genes were STATc itself, the STATc inhibitor PTP3, and five genes encoding TKL proteins including the DIF-regulated STATc activator Pyk2 (Table 3). We confirmed their differential regulation by real time PCR by comparing the expression profiles of treated versus untreated Ax2 wild-type and STATc^−^ cells (Fig. 1). All of these genes showed significant upregulation upon treatment with sorbitol in Ax2 wild-type cells which was lost in the STATc^−^ strain.

**Table 3:** Selection of STATc regulated genes.

dictyBase ID	Gene Name	Gene Product
DDB_G0293532	***dstC***	signal transducer and activator of transcription (STAT) family protein DstC ( = STATc)
DDB_G0282145	***ptpC***	protein tyrosine phosphatase PTP3
DDB_G0283385	***pyk1***	Pyk1/SplA tyrosine kinase-like protein (TKL group)
DDB_G0285321	***pyk2***	Pyk2/SplB tyrosine kinase-like protein (TKL group)
DDB_G0289001	***pyk3***	Pyk3/PkyA tyrosine kinase-like protein (TKL group)
DDB_G0283699	***phg2***	Phg2 tyrosine kinase-like protein (TKL group)
DDB_G0282895	***DDB_G0282895***	MORN repeat-containing protein, tyrosine kinase-like protein (TKL group)

Ax2 and STATc^−^ cells were treated for 15 min with 200 mM sorbitol or left untreated and differentially regulated genes were identified by microarray analysis [2]. Depicted are *dstc*, *ptpc* and five genes encoding TKL proteins.

The *D. discoideum* genome does not encode an obvious ortholog of the mammalian JAKs and the question was, whether the microarray and real time PCR results could point us to upstream regulators of STATc. The upregulation of STATc, PTP3, and Pyk2 upon sorbitol treatment suggested the existence of a positive feedback loop also for other genes encoding proteins of the STATc signalling cascade. Based on the assumption of i) a positive transcriptional feedback loop, ii) their STATc-dependent differential regulation, and iii) the presence of a tyrosine-kinase-like catalytic domain, we considered Pyk1, Pyk3, Phg2, and the MORN kinase as potential upstream regulators of STATc. The Pyk2 kinase was excluded from further analysis because its genetic ablation has been shown to cause only a one minute delay in sorbitol-induced STATc activation [11]. BLAST searches against the *D. discoideum* proteome with a human JAK protein sequence (NP_002218) revealed highest sequence similarity with Pyk3. Furthermore, it was previously shown that Phg2 interacts with Rap1-GTP which has been linked to the response to sorbitol [23]. We therefore decided to further analyse the cellular functions of Pyk3 and Phg2 with respect to STATc signalling. They encode TKL proteins of 1338 and 1387 amino acids, respectively. Pyk3 contains a long N-terminal region of mostly low compositional complexity and two C-terminal protein kinase domains, of which the first one is predicted to be a pseudokinase as is the case for members of the JAK family (Fig. S2). In contrast, Phg2 has an N-terminal coiled coil region followed by a ras binding domain (RBD, aa 594–667), a protein kinase domain (aa 802–1074), and long stretches of low compositional complexity in the remaining sequence (Fig. S2; [23]). We generated knock-out mutants for Pyk3, Phg2, and a double knock-out for both genes and verified them by reverse transcription (RT) PCR and Western blot analysis (Fig. S1 and S3). An independent pyk3^−^ knock-out strain was generated by Tsuyoshi Araki (University of Dundee). We exchanged the two Pyk3 knock-out mutants and in all experiments where we used them in parallel we obtained similar results (data not shown).

### Pyk3 and Phg2 are Part of the STATc Signalling Cascade

**Figure 2:**
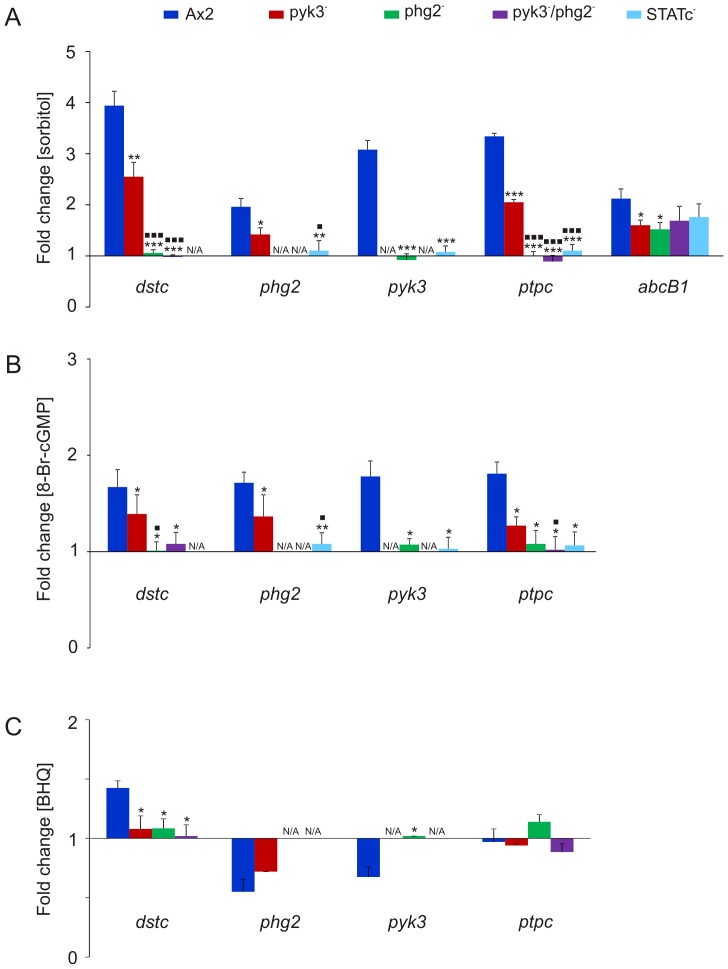
Pyk3 and Phg2 are required for full transcriptional activation of STATc-dependent genes. Real time PCR analysis of selected genes was carried out with cDNAs from untreated cells and from cells treated for 15 minutes with either 200(A), 20 mM 8-Br-cGMP (B) or 30 µM BHQ (C). The differential expression was investigated in Ax2 wild-type, pyk3^−^, phg2^−^, pyk3^−^/phg2^−^, and STATc^−^ cells upon treatment with sorbitol and 8-Br-cGMP (A, B) or in Ax2 wild-type, pyk3^−^, phg2^−^, and pyk3^−^/phg2^−^ cells upon treatment with BHQ (C). The data are expressed as means of fold change in comparison to untreated cells. A students t-test was performed and significance values were calculated: p-value <0.05 = */^▪^; p-value <0.01 = **/^▪▪^_;_ p-value <0.001 = ***/^▪▪▪^_._ Fold changes and standard deviations of six measurements from three independent experiments are shown. *Statistically significant changes, compared with AX2 cells. **^▪^**Statistically significant changes, compared with pyk3^−^ cells. N/A: *not applicable.*

We first investigated whether Pyk3 and/or Phg2 are required for stress-induced transcription of STATc-regulated genes. Ax2 wild-type, the STATc^−^, pyk3^−^, phg2^−^, and pyk3^−^/phg2^−^ strains were treated with 200 mM sorbitol or left untreated, RNA was isolated, reverse transcribed, and quantitative real time PCR was performed for *dstc*, *phg2*, *pyk3*, *ptpc,* and *abcB1* (ABC transporter B family protein) as control. *abcB1* was previously found to be differentially regulated independently of STATc in response to osmotic stress [2]. All of these genes except *abcB1* were upregulated in Ax2 wild-type cells but not differentially regulated in the STATc^−^ cells. This is consistent with previous microarray and quantitative real time PCR results (Table 3, Fig. 1, [2]). In contrast, the sorbitol-induced upregulation of all selected genes was approximately two-fold lower in the pyk3^−^ cells indicating the need for Pyk3 for a full transcriptional response to sorbitol. In phg2^−^ and pyk3^−^/phg2^−^ cells the sorbitol-induced expression of the selected genes was lost. This showed that the presence of Phg2 is essential for the transcriptional regulation of these STATc dependent genes (Fig. 2A).

It has been previously shown that two signalling branches converge on STATc in response to hypertonicity, which are under control of the second messengers cGMP and Ca^2+^, respectively [7]. We therefore treated the cells with 8-Br-cGMP, a membrane-permeable cGMP analogue and a known activator of STATc [3], [24]. The results were principally similar to the sorbitol treatment, however, the overall fold changes for Ax2 and pyk3^−^ cells were generally lower except for the upregulation of *phg2* indicating that the activation of the cGMP branch is not sufficient for full transcriptional activation (Fig. 2B). We next investigated the role of Ca^2+^ in the differential regulation of these genes by treating Ax2 wild-type, pyk3^−^, phg2^−^, and pyk3^−^/phg2^−^ cells with tBuBHQ (BHQ), which causes an increase in cytosolic Ca^2+^ levels. The real time PCR results showed that only *dstc* but not the other genes were upregulated under these conditions in Ax2 wild-type cells, but not in the mutants (Fig. 2C). The fold changes were again, as with 8-Br-cGMP, significantly lower when compared to the response to sorbitol. In addition, we found a down-regulation for *phg2* in Ax2 and pyk3^−^ cells and for *pyk3* in Ax2 wild-type cells. We obtained similar results when we treated the cells with thapsigargin (data not shown). In summary, these results show that activation of both, the cGMP and the Ca^2+^, branches are needed for a full transcriptional response of STATc and the investigated STATc dependent genes. They also show that expression of STATc can be induced by a rise in cGMP as well as Ca^2+^, while induction of *phg2*, *pyk3* and *ptp3* needs activation of the cGMP branch. Interestingly, the observed fold changes in response to sorbitol in AX2 cells were generally higher than the sum of these changes in response to 8-Br-cGMP and BHQ. This is very obvious for *pyk3* where we see a smaller upregulation in response to 8-Br-cGMP as compared to sorbitol and a downregulation in response to BHQ. The discrepancy in the fold change in response to sorbitol versus activation of the single branches can be explained if we assume a crosstalk between the two branches where e.g. activation of the cGMP branch would somehow exert an inhibitory effect on the Calcium branch. Alternatively, sorbitol might activate yet another, so far unknown signalling pathway.

### STATc Phosphorylation is Reduced in pyk3**^−^**, phg2**^−^** and pyk3**^−^**/phg2**^−^** Cells

**Figure 3:**
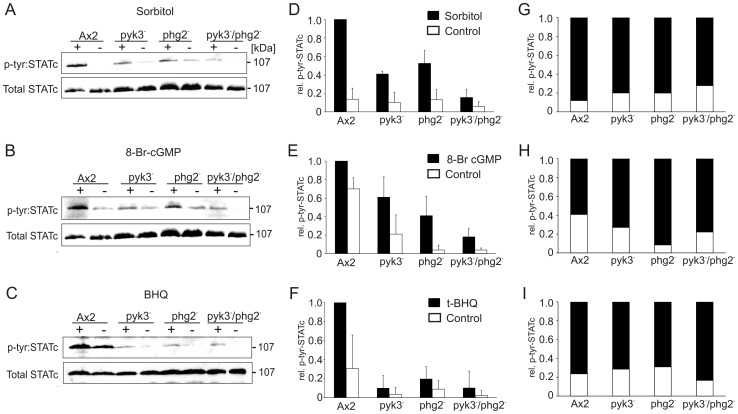
Phosphorylation of STATc is reduced in the absence of Pyk3 and Phg2. Ax2 wild-type, pyk3^−^, phg2^−^, and pyk3^−^/phg2^−^cells were either treated (+) for 15 minutes with 200 mM sorbitol (A), 20 mM 8-Br-cGMP (B), 30 µM BHQ (C) or left untreated (−). Total cell lysates were prepared, proteins were separated by 10% SDS-PAGE and transferred to nitrocellulose. Total and tyrosine phosphorylated STATc were detected with the CP22 and 7H3 antibodies, respectively. (D–F): Quantification of phosphorylated STATc in either treated or untreated cells. Band intensities were determined densitometrically and normalised using the values for total STATc. The amount of phosphorylated STATc of treated Ax2 cells was set to 1 and relative values were calculated for untreated Ax2 cells as well as treated and untreated mutants. The error bars depict standard deviations of three (D, E) and six (F) independent experiments. (G–I): Staple diagrams depicting the relative amount of phosphorylated STATc from treated (black part) versus untreated (white part) cells. The total amount of phosphorylated STATc was set to 1 for each comparison and relative values were calculated.

When *D. discoideum* cells are exposed to 200 mM sorbitol STATc is activated by phosphorylation on Tyr922. In the absence of hyperosmotic conditions STATc phosphorylation can also be induced by 8-Br-cGMP and BHQ [3]. We tested the potential involvement of Pyk3 and Phg2 in STATc activation by analysing STATc phosphorylation levels on Tyr922 in Ax2, pyk3^−^, phg2^−^, and pyk3^−^/phg2^−^ cells. For quantitation of phospho- and total STATc we used the STATc phosphorylation-specific monoclonal antibody CP22 and the total STATc antibody 7H3 [10]. Cells were treated with either sorbitol or 8-Br-cGMP or BHQ and Western Blot analyses were performed. In Ax2 cells, we observed a strong induction of STATc phosphorylation upon sorbitol treatment. In contrast, pyk3^−^ and phg2^−^ cells showed significantly lower levels of phosphorylated STATc and the pyk3^−^/phg2^−^ double mutant displayed a further decrease but not a complete abolishment of STATc phosphorylation (Fig. 3A). Densitometry analyses of three independent experiments revealed in the single mutants an approximately two-fold and in the double mutant an approximately five-fold lower level of STATc phosphorylation (Fig. 3D; STATc phosphorylation of sorbitol treated Ax2 cells is set to 1). Next we stimulated the different strains with 8-Br-cGMP and obtained similar results as upon sorbitol treatment. Specifically, we observed an approximately two-fold reduction of p-STATc in the single mutants and an approximately five-fold reduction in the double mutant. For all strains we found a clear induction of STATc phosphorylation upon treatment though the level of p-STATc was in this series of experiments already quite high in untreated Ax2 cells (Fig. 3B and E). To investigate whether a rise in intracellular Ca^2+^ could induce STATc phosphorylation in Ax2 and the mutant strains we treated the cells with BHQ. We observed a clear induction of STATc phosphorylation in Ax2 cells. In comparison to Ax2 total p-STATc levels were extremely low in pyk3^−^, phg2^−^, and pyk3^−^/phg2^−^ in response to BHQ, however, phosphorylation of STATc still appeared to be induced upon treatment (Fig. 3C and F). It should be noted that, although absolute p-STATc levels upon treatment were always significantly lower in the mutant strains as compared to wild-type, treatment with either sorbitol or 8-Br-cGMP or BHQ still appeared to induce phosphorylation for STATc in all strains. A measure of the induction is the ratio of p-STATc in treated versus untreated cells. It clearly shows that for all strains phosphorylation of STATc is induced for all three conditions (Fig. 3G–I). However, it is at present not possible to make a firm statement if in addition to the absolute levels also the relative induction of STATc phosphorylation was changed in the mutant strains because the standard deviations of the individual experiments were rather high. In summary we conclude from these results that Pyk3 and Phg2 somehow contribute to STATc phosphorylation in response to hyperosmotic conditions. The further decrease of STATc phosphorylation in the double mutant in comparison to the single knock-out mutants in response to sorbitol and 8-Br-cGMP suggests that Pyk3 and Phg2 act in parallel.

### Nuclear Translocation of STATc is Delayed in Mutant Strains

**Figure 4:**
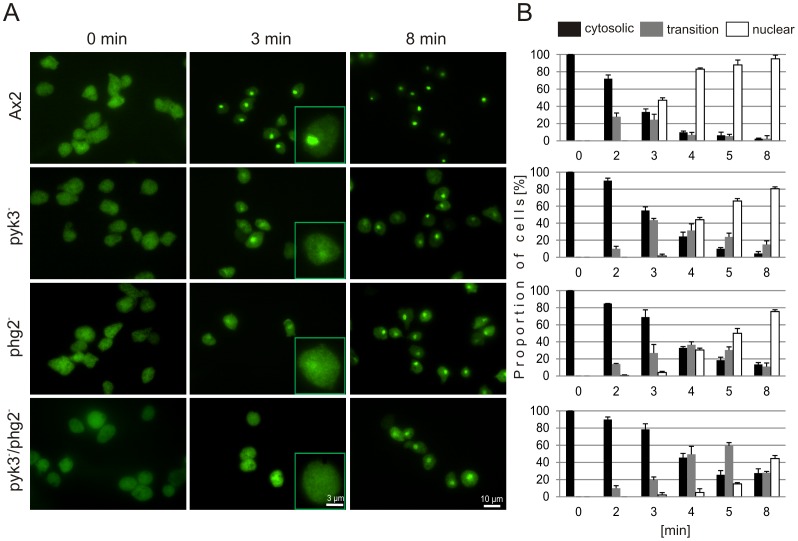
Nuclear translocation of STATc is delayed in pyk3^−^, phg2^−^, and pyk3^−^/phg2^−^ cells. (A) Ax2 wild-type, pyk3^−^, phg2^−^, and pyk3^−^/phg2^−^ cells expressing GFP-STATc were treated with 100 mM sorbitol for the indicated times to induce GFP-STATc nuclear translocation, fixed with ice-cold methanol, and observed under the fluorescence microscope. Exemplary images of GFP-STATc expressing cells after 0, 3, and 8 min of treatment are shown and the insets at 3 min show a single exemplary cell for each strain to show prominent nuclear (Ax2), transition i.e. beginning nuclear (pyk3^−^ and phg2^−^) and clear cytosolic (pyk3^−^/phg2^−^) localisation of GFP-STATc. (B) Quantification of GFP-STATc nuclear translocation in Ax2 wild-type and mutant cells. For each time point, we analysed 150 cells per experiment and determined the number of cells showing either clear cytosolic (black bar), transition i.e. beginning nuclear (grey bar) or prominent nuclear (white bar) localisation of GFP-STATc. The percentage of cells in each of these three categories was calculated. Error bars depict standard deviations of two independent experiments.

Hyperosmotic conditions trigger STATc phosphorylation and dimerization followed by nuclear translocation enabling STATc to differentially regulate the expression of a large number of target genes [2], [3]. We expressed GFP-STATc in Ax2 wild-type, pyk3^−^, phg2^−^, and pyk3^−^/phg2^−^ cells. Western blot analysis using a specific monoclonal GFP antibody showed that all strains expressed GFP-STATc at similar levels, with exception of the pyk3^−^/phg2^−^ strain where GFP-STATc levels were lower (Fig. S4). Nuclear translocation of GFP-STATc was induced by treatment with sorbitol for different time intervals and analysed microscopically. Assessment of GFP-STATc nuclear translocation after 3 and 8 minutes of treatment indicated a delay in the mutant strains (Fig. 4A). Quantitative analyses of two independent experiments clearly confirmed this phenotype. For example at the three minutes time point GFP-STATc is found in the nucleus in about 50% of Ax2 wild-type cells. In contrast, in the pyk3^−^ and phg2^−^ mutants full nuclear translocation of GFP-STATc was only seen in around 1–5% of the cells. For around 30–50% of the cells we observed partial translocation and in the remaining 50–70% GFP-STATc was still completely cytosolic. The delay was even more pronounced in the pyk3^−^/phg2^−^ cells as GFP-STATc was still found in the cytosol in almost 80% of the cells at this time point (Fig. 4B). Nuclear translocation of GFP-STATc in the three mutant strains was also delayed at the other time points (Fig. 4B; Fig. S5). The results show that Pyk3 and Phg2 are required for efficient nuclear translocation of STATc in response to sorbitol. The further delay of nuclear translocation of STATc in the double mutant in comparison to the single knock-out mutants again suggests that Pyk3 and Phg2 act in parallel.

### Phg2 but not Pyk3 is Upstream of PTP3

**Figure 5:**
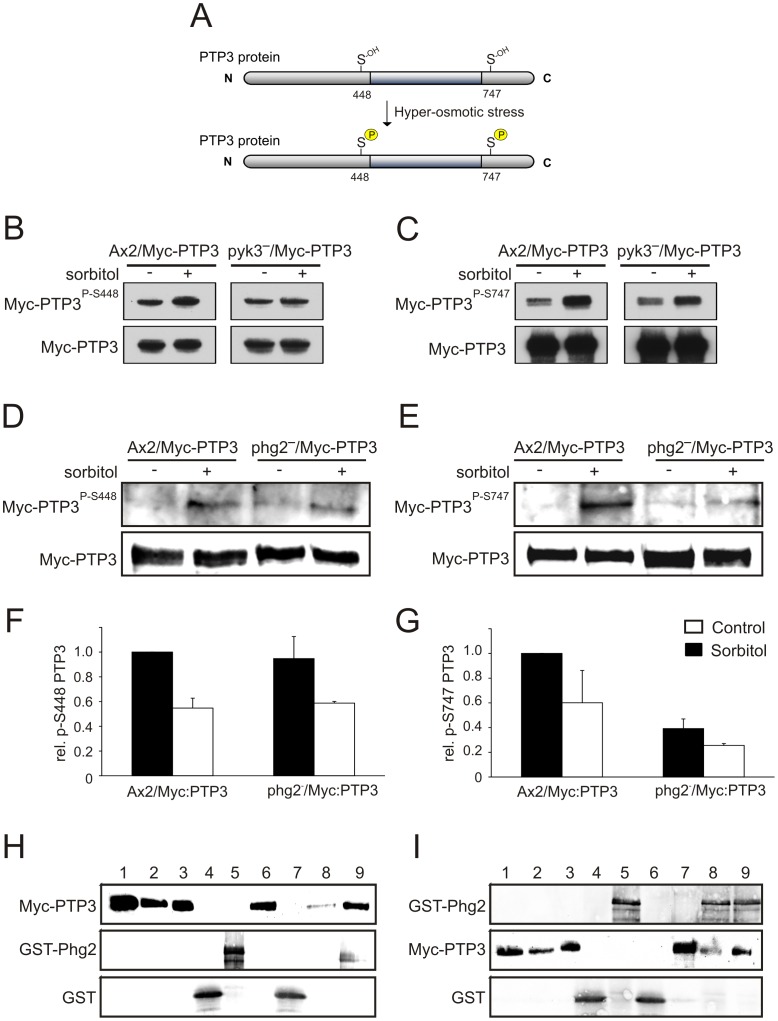
Phg2 but not Pyk3 acts upstream of PTP3. (A) Upon hyperosmotic conditions, PTP3 becomes phosphorylated on two serine residues, S448 and S747, resulting in an inhibition of its phosphatase activity [7]. (B–E) Ax2/Myc-PTP3, pyk3^−^/Myc-PTP3 and phg2^−^/Myc-PTP3 cells were left untreated (−) or treated (+) with 200 mM sorbitol for 5 min (B, C) or 10 min (D, E). Myc-PTP3 was immunoprecipitated, separated by SDS-PAGE, transferred to nitrocellulose, and phosphorylated Myc-PTP3 was detected with PTP3 antibodies specific for phospho-serine 448 (B, D) and phospho-serine 747 (C, E). Total Myc-PTP3 was used as loading control and detected with an anti-Myc antibody (mAb 9E10). (F, G): Quantification of PTP3 phospho-serine 448 (F) and 747 (G) in either treated or untreated cells. Band intensities were determined densitometrically and normalised using the values for total Myc-PTP3. The amount of serine phosphorylated PTP3 of treated Ax2/Myc-PTP3 cells was set to 1 and relative values were calculated for untreated Ax2/Myc-PTP3 cells as well as for treated and untreated phg2^−/^Myc-PTP3 cells. The error bars depict standard deviations of three independent experiments. (H, I): Pull-down assay to investigate the binding of Myc-PTP3 to bacterially expressed GST-Phg2. In the first approach (H) Myc-PTP3 was expressed in phg2^−^ cells, immunoprecipitated with anti-Myc Dynabeads, and eluted from the beads via a pH shift for binding to GST-Phg2 bound to glutathione beads. Lane 1: Total cell lysate of Myc-PTP3 expressing phg2^−^ cells; lane 2: Anti-Myc Dynabeads after immunoprecipitation of Myc-PTP3; lane 3: Myc-PTP3 after elution from anti-Myc Dynabeads; lane 4: GST, purified from bacteria and bound to glutathione-beads (GST-beads); lane 5: GST-Phg2, purified from bacteria and bound to glutathione-beads (GST-Phg2-beads); lane 6: supernatant after incubation of Myc-PTP3 with GST-beads; lane 7: pellet after incubation of Myc-PTP3 with GST-beads; lane 8: supernatant after incubation of Myc-PTP3 with GST-Phg2-beads; lane 9: pellet after incubation of Myc-PTP3 with GST-Phg2-beads. In the reverse approach (I) the binding of bacterially expressed GST-Phg2, which was eluted from glutathione-beads, to Myc-PTP3 bound to anti-Myc Dynabeads (Myc-PTP3-beads) was investigated. Lane 1: Total cell lysate of Myc-PTP3 expressing phg2^−^ cells; lane 2: lysate after immunoprecipitation of Myc-PTP3 with anti-Myc Dynabeads; lane 3: Anti-Myc Dynabeads with bound Myc-PTP3; lane 4: GST, purified from bacteria and eluted from glutathione-beads; lane 5: GST-Phg2, purified from bacteria and eluted from glutathione-beads; lane 6: supernatant after incubation of GST with Myc-PTP3-beads; lane 7: pellet after incubation of GST with Myc-PTP3-beads; lane 8: supernatant after incubation of GST-Phg2 with Myc-PTP3-beads; lane 9: pellet after incubation of GST-Phg2 with Myc-PTP3-beads. The order of lines from the same immunoblot were digitally re-arranged for illustration purposes to omit dispensable lines.

The protein tyrosine phosphatase PTP3 is crucial for the balance between phosphorylated and dephosphorylated STATc. PTP3 is constitutively active in its non-phosphorylated form and removes the phosphate from Tyr922 of STATc. Upon hyperosmotic stress, PTP3 becomes phosphorylated on serines 448 and 747 (Fig. 5A) [7]. This results in a decrease of its enzymatic activity and shifts the balance to tyrosine phosphorylation and activation of STATc [7], [12], [13]. Our results so far show that Pyk3 and Phg2 are part of the STATc signalling cascade. Their cellular functions could be exerted by either positively influencing STATc phosphorylation or by negatively acting on PTP3.

To directly study phosphorylation of PTP3 on S448 and S747 we generated Myc-PTP3 overexpressing Ax2 wild-type, pyk3^−^ and phg2^−^ cells, treated them with sorbitol, and performed Western blot analyses using antibodies specific for phospho-S448 and phospho-S747 PTP3, respectively [7]. In comparison to Ax2/Myc-PTP3 cells sorbitol-treatment of pyk3^−^/Myc-PTP3 cells resulted in Myc-PTP3 S448 and S747 phosphorylation (Fig. 5B, C). The absolute level of phosphorylated S747 was slightly and ofr S448 considerably lower in pyk3^−^/Myc-PTP3 cells in response to sorbitol indicating that Pyk3 may somehow contribute to full inhibition of PTP3. To further investigate the involvement of Pyk3 in the phosphorylation of S448 of PTP3 we expressed GST-PTP3ΔCS [12] in bacteria and performed *in vitro* phosphorylation assays using the 50% NH_4_SO_4_ soluble protein fraction from Ax2 and pyk3^−^ cells. We found that both lysates contain an activity that is induced by sorbitol and phosphorylates S448 of PTP3 (data not shown). These results exclude Pyk3 as a specific inhibitor of PTP3 and suggest that it is involved in the phosphorylation of STATc as a regulated activator. This could in principle be analysed using an immunoprecipitation-kinase assay but, given that any difference might be expected to be only two-fold, it would be a difficult task.

Next we analysed the possible involvement of Phg2 in the modulation of STATc activity by inhibition of PTP3. As described above for Pyk3, Myc-PTP3 overexpressing Ax2 wild-type and phg2^−^ cells were treated with sorbitol and Western blot analyses using the phospho-specific PTP3 antibodies were performed. We observed a similar increase in Myc-PTP3 S448 phosphorylation in both strains upon sorbitol treatment (Fig. 5D) which was confirmed by densitometric quantitation of three independent experiments (Fig. 5F). In contrast, Myc-PTP3 S747 phosphorylation was nearly completely abolished in the phg2^−^ strain (Fig. 5E). Quantitation as for the PTP3 S448 specific phosphorylation revealed a strongly reduced signal in the phg2^−^ strain which was about three fold weaker as in the Ax2 strain (Fig. 5G). The remaining weak signal in the phg2^−^ strain could be caused by a PTP3 S747 specific protein kinase which is either downstream of Phg2 or acts in parallel to Phg2 or could be due to cross-reactivity of the PTP3 S747 phospho-specific antibody with non-phosphorylated PTP3. At present we cannot exclude any of these possibilities. These results also show that at least two distinct serine/threonine kinases are responsible for the phosphorylation of PTP3 at S448 and S747.

To investigate a possible direct interaction between Phg2 and PTP3 we performed pull-down experiments using immunopurified Myc-PTP3 from phg2^−^ cells and GST-Phg2 purified from bacteria. The assay was performed with either eluted Myc-PTP3 and GST-Phg2 bound to glutathione beads (Fig. 5H) or Myc-PTP3 bound to beads and eluted GST-Phg2 (reverse approach, Fig. 5I). The pull-down experiments showed for both approaches *in vitro* binding of Phg2 to PTP3. In the first approach where GST-Phg2 was bound to glutathione beads most of Myc-PTP3 was found in the pellet fraction and only a small fraction remained in the supernatant (Fig. 5H, lanes 8 and 9). In the reverse approach Myc-PTP3 bound to anti Myc Dynabeads pulled down GST-Phg2 (Fig. 5I, lanes 8 and 9). Soluble GST and GST bound to beads, respectively, were used as negative controls. Neither Myc-PTP3 bound to GST beads (Fig. 5H, lanes 6 and 7) nor GST bound to Myc-PTP3 beads (Fig. 5I, lanes 6 and 7). The pull-down results demonstrate a direct interaction between Myc-PTP3 purified from phg2^−^ cells and recombinant GST-Phg2 purified from *E. coli*.

## Discussion

STATc, one of four STAT proteins in *D. discoideum*, is responsible for a significant part of the transcriptional changes in response to hyperosmotic conditions [2]. STATc becomes tyrosine phosphorylated on Tyr922 and nuclear enriched by stress insults, but also in response to the chlorinated hexaphenone DIF-1 during development [3], [10]. The activation of metazoan STAT proteins is generally mediated by members of the Janus tyrosine Kinase (JAK) protein family, whose activity is regulated by growth hormones and cytokines [25]. Binding of a cytokine to its cognate tyrosine kinase cell surface receptor triggers autophosphorylation of the specific receptor-bound JAK. The activated JAK phosphorylates a tyrosine residue of the receptor that serves as a docking site for the STAT SH2 domain. The JAK then phosphorylates the bound STAT at a specific tyrosine close to its C-terminus. This triggers homo- or hetero-dimerization and nuclear translocation of the STATs where they control the expression of a large number of target genes [5], [26]. However, the *D. discoideum* genome does not encode bona fide tyrosine kinases and there is no JAK ortholog. Tyrosine phosphorylation in *D. discoideum* appears to be mediated by a subset of the Tyrosine Kinase Like (TKL) proteins of which 66 are encoded in the genome [6]. The catalytic sequence motifs of the TKL protein kinases resemble both, tyrosine and serine/threonine kinases. In our microarray analysis of Ax2 wild-type and STATc^−^ cells five genes encoding TKLs were already differentially regulated 15 min after exposure to sorbitol and their differential expression was dependent on the presence of STATc (Table 3; [2]). Based on the assumption of a positive feedback loop in the STATc signalling cascade we considered these TKLs as potential upstream regulators of STATc. Notably, the gene encoding the TKL Pyk2 is among these STATc dependent early differentially regulated genes. Pyk2 was recently shown to form a complex with STATc *in vitro* and *in vivo* and to directly phosphorylate it. As proposed by Araki et al. Pyk2 is constitutively active and there is parallel inhibition of the cognate tyrosine phosphatase PTP3 by phosphorylation of two serine residues, S448 and S747 [7], [11].

Our work aimed at the identification of protein kinases apart from Pyk2, which are involved in STATc regulation. Based on the early differential regulation in our microarray approach, their suspected catalytic activity and previously published results, we chose Pyk3 and Phg2 for further investigation. Pyk3 was originally identified by screening of a λgt11 expression library with a phospho-tyrosine specific antibody [23]. More recently it was found, that in contrast to wild-type cells where STATc phosphorylation in response to DIF-1 peaked after about 3 minutes, Pyk3 knock-out cells displayed persistent STATc phosphorylation which correlated with aberrant pattern formation during development. The authors propose that Pyk3 acts upstream of PTP3 but do not present experimental evidence [27]. Contrary to this hypothesis we demonstrate that phosphorylation on S448 and S747 of PTP3 does not depend on Pyk3 and that its knock-out results in only 50% lower levels of STATc phosphorylation and a delay in its nuclear translocation in response to sorbitol as compared to wild-type cells (Figs. 3, 4, 5). The induction of STATc and STATc dependent genes was also reduced by only about 50% in pyk3^−^ cells (Fig. 2). Our results are consistent with a model wherein Pyk3 does not inhibit PTP3 but rather directly or indirectly activates STATc. The remaining lower level of STATc phosphorylation in the pyk3^−^ strain is we believe due to the activity of Pyk2 (Fig. 3; [11]; Araki et al., unpublished). The presence of reduced levels of STATc phosphorylation in the pyk3^−^ strain in response to either sorbitol, 8-Br-cGMP, or BHQ can be explained if one assumes that i) Pyk2 is either constitutively active, as proposed by Araki et al [11], or can also (in addition to DIF-1) be activated through the cGMP branch and that ii) PTP3 is parallelly inhibited. Our results are not in agreement with the mode of Pyk3 action proposed by Lee et al. [27]. The major difference between the two studies is that Lee et al. investigated development and the DIF-1 response in their pyk3^−^ strain in an Ax4 background [27] while we analysed the response to hyperosmotic conditions in an Ax2 background.

Phg2 belongs to the TKL group but the recombinant kinase domain was shown to phosphorylate serine and threonine in vitro. The gene was originally found to be disrupted in a REMI screen for phagocytosis deficient mutants and the recapitulated knock-out mutant displayed in addition defects in cellular adhesion, cell motility, and actin organization [23]. It was also found that Phg2 is a Rap1 effector and directly interacts with Rap1 via a ras-binding domain (RBD) in the protein sequence and that the protein binds PI(4,5)P_2_ via a novel binding site, which mediates membrane localization [23], [28], [29]. Rap1 is implicated in chemotaxis, cell adhesion and cell polarity and also in the hyperosmotic response and thought to activate different effectors [30]–[32]. Phg2 was identified as a Rap1 effector necessary for cell adhesion but not cell polarity [29]. We found that in response to hyperosmolarity Phg2 affects STATc phosphorylation and nuclear accumulation (Figs. 3, 4, S5). Furthermore, overexpression of Phg2 in wild-type cells caused an increase in STATc protein levels (Fig. S6), and in real time PCR analysis we found that the upregulation of STATc in response to sorbitol and 8-Br-cGMP is completely dependent on Phg2 (Fig. 2). Notably, in the phg2^−^ mutant STATc still became activated and translocated to the nucleus, albeit with a significant delay (Figs. 3, 4). We propose that Phg2 is required for the nuclear translocation of an additional protein that is needed for the transcriptional activity of STATc. Further work is needed to elucidate the identity of this factor. Another result of the real time PCR analysis was that the fold changes of the differentially regulated genes were always lower in response to 8-Br-cGMP and BHQ than to sorbitol (Fig. 2). This result suggests that activation of both the cGMP and the Ca^2+^ branch of STATc signalling is needed for a full transcriptional response. In previous studies Phg2 had not been implicated in STATc signaling and we show for the first time that it also plays an important role in the hyperosmotic response. Its phosphorylation specificity for Ser/Thr excluded STATc^Tyr922^ as target [23]. Therefore, we investigated if Phg2 could possibly act on PTP3, the constitutive active tyrosine phosphatase which counterbalances STATc phosphorylation [7]. Using phosphorylation specific antibodies we provide evidence that phosphorylation of PTP3^S747^ but not PTP3^S448^ is significantly reduced in the phg2^−^ mutant (Fig. 5D, E, F, G). Furthermore, we could show that myc-PTP3 from phg2^−^ cells pulled down recombinant GST-Phg2 and vice versa (Figs. 5H, I). However, and despite several attempts, we could so far not directly phosphorylate PTP3 with Phg2. Thus, Phg2 might activate a downstream protein kinase which could act on PTP3. Alternatively, it is possible that recombinant Phg2 is enzymatically not active but nevertheless able to strongly bind PTP3 and that we need an additional factor, as for example active Rap1, to activate the protein kinase. Further work is needed to clarify this issue.

**Figure 6:**
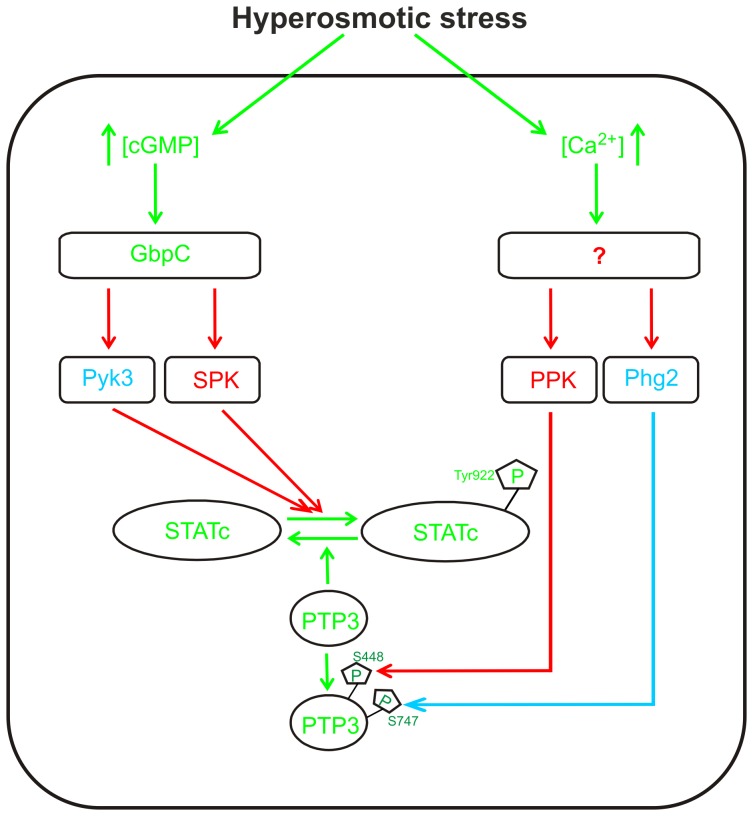
Model of STATc activation in response to hyperosmotic conditions. The model is based on results from previously published studies (green) and from results (blue) and educated assumptions (red) of the present work. In response to hyperosmolarity intracellular cGMP and Ca^2+^ act in parallel as second messengers in order to activate STATc. GbpC is downstream of cGMP and we postulate that it acts upstream of Pyk3 and an as yet unidentified STATc protein kinase (SPK). Phg2 inhibits PTP3 either directly or indirectly by phosphorylation of S747 and an unknown additional PTP3 serine/threonine protein kinase (PPK) must be responsible for phosphorylation of PTP3 at S448. These protein kinases are under control of the Ca^2+^ branch of the STATc signaling cascade [7]. The model does not satisfactorily explain all experimental results and we additionally propose a crosstalk between the two signaling branches (not shown). See the Discussion section for further details of this model.

In conclusion, our study provides additional insights into the STATc activation pathway in *D. discoideum* that are illustrated in a flow chart (Fig. 6). This figure depicts a possible STATc activation mechanism, which is based on published results (green), results (blue) and educated assumptions (red) from the present study. STATc becomes activated upon hyperosmolarity via two signaling branches, the cGMP-dependent and the Ca^2+^-dependent branch, and an increase in intracellular cGMP levels activate GbpC in the cGMP branch [7]. The downstream target of Ca^2+^ is not yet known. Recently it was shown that phosphorylation of PTP3^S747^ and PTP3^S448^ could not be triggered by cGMP but was dependent on Ca^2+^ [7]. We provide evidence that Phg2 acts upstream of PTP3 and either directly phosphorylates S747 or activates another downstream protein kinase which then phosphorylates S747. Phosphorylation of PTP3^S448^ was unchanged in the phg2^−^ mutant and we could show that Pyk3 is not responsible for it (Fig. 5). Therefore, the S448 phosphorylation must be accomplished by an as yet unidentified PTP3 ser/thr protein kinase (PPK). As a consequence PTP3 is only partially inhibited in response to sorbitol in the phg2^−^ mutant which explains the reduced STATc phosphorylation in this mutant. The fact that the phenotype of the pyk3^−^/phg2^−^ double mutant was in all our experiments more severe than either of the single mutants supports the hypothesis that neither Pyk3 acts upstream of Phg2 nor vice versa, but that both proteins act in parallel to modulate STATc (Figs. 3–5). We have obtained reduced STATc phosphorylation in two independently generated pyk3^−^ mutants (labs of JGW and LE, not shown). Therefore, we postulate that the reduced level of p-STATc in the pyk3^−^ strain in response to sorbitol and 8-Br-cGMP but not BHQ is due to the presence of another, as yet unidentified, STATc protein kinase (SPK) downstream of cGMP. The exact target and action of Pyk3 has still to be elucidated and STATc is an attractive candidate. The STATc phosphorylation patterns (Fig. 3) can be explained with Pyk3 acting downstream of cGMP and in parallel to the postulated SPK. This requires that the activity of the as yet unknown SPK is induced via cGMP but not via the Ca^2+^ liberator BHQ. It is of particular interest, that the TKL Pyk2 was recently shown to directly phosphorylate STATc in response to DIF-1 [11]. Thus, it is possible that the reduced level of p-STATc in the pyk3^−^ strain in response to sorbitol and 8-Br-cGMP but not BHQ is due to Pyk2. It should be noted that the model does not fully explain all details of our results, rather it presents a simplified working hypothesis that explains the major aspects of STATc regulation in response to hyperosmolarity. As hypothesized for the transcriptional regulation (Fig. 2) we think that subtle discrepancies in STATc phosphorylation in response to sorbitol, 8-Br-cGMP or BHQ (Fig. 3) to expected changes are due to cross talk between the two branches. Alternatively, another pathway might be activated by sorbitol in addition to cGMP and Ca^2+^. Future work will address these possibilities and elucidate additional components of the complex *D. discoideum* STATc signaling cascade that are together responsible for optimal STATc activation in response to different cues.

## Supporting Information

Figure S1 **Generation of knock-out mutants.** (A) The phg2^−^ strain was generated by replacement of a 2.9 kbp genomic fragment of the *phg2* gene that contained part of the tyrosine kinase domain coding sequence with the targeting construct containing the blasticidin resistance cassette flanked by *loxP* recombination sites. The targeting construct with the blasticidin resistance cassette is shown on top, the genomic sequence of *phg2* before the recombination event in the middle, and after the recombination event at the bottom. BamHI, PstI, HindIII, SalI: DNA restriction enzyme sites that were used for the generation of the targeting construct. A8P: Actin 8 promoter. A15T: Actin 15 terminator. Bsr: blasticidin resistance gene. The illustration does not reflect the true scale of actual parts of the disruption vector. (B) The pyk3^−^ strain was generated by replacement of a 2.37 kbp genomic fragment of the *pyk3* gene with the targeting strategy as described for phg2^−^. The pyk3^−^/phg2^−^ double knock-out strain was generated by transforming the pyk3^−^ strain after transient expression of the *cre recombinase* (restored blasticidin sensitivity) with the targeting construct for *phg2* (shown at the bottom).
(PDF)

Figure S2 **Domain structure of** ***D. discoideum*** **Pyk3 and Phg2.** The protein sequences of Pyk3 (DDB_G0289001) and Phg2 (DDB_G0283699) were analysed with SMART (http://smart.embl-heidelberg.de/) to elucidate their domain architecture. STYKc: possible dual-specificity Ser/Thr/Tyr protein kinase domain; green rectangles: coiled coil regions; lilac rectangles: segments of low compositional complexity; aa: amino acids.
(PDF)

Figure S3 **Analysis of the generated pyk3^−^, phg2^−^ and pyk3^−^/phg2^−^ knock-out strains.** (A) Reverse transcription PCR using primer pairs that amplify small fragments of the 3′-end of the *pyk3*, *phg2,* and *dstc* cDNA, respectively. *dstc* was used as a positive control. (B) Western blot analysis of total cell lysates of Ax2 wild-type, pyk3^−^, phg2^−^, and pyk3^−^/phg2^−^ cells. Proteins were separated by SDS-PAGE, transferred to nitrocellulose and Western blot analyses with polyclonal pyk3 and phg2 antibodies were performed. Severin was used as loading control and detected with a monoclonal severin antibody (mAb 101-460-2). Please note that the weak band in the pyk3^−^ strain (*) is due to cross-reaction of the Pyk3 antibody. A similar cross-reaction with this antibody was also seen in the independently generated pyk3^−^ mutant in the lab of J.G. Williams (data not shown).
(PDF)

Figure S4 **Western Blot analysis of GFP-STATc expression in Ax2, pyk3^−^, phg2^−^, and pyk3^−^/phg2^−^ cells.** The expression of GFP-STATc was monitored by Western blot analysis of total cell lysates with a monoclonal GFP antibody (mAb K3-184-2). Actin was used as loading control and detected with a monoclonal actin antibody (mAb Act1-7).
(PDF)

Figure S5 **Immunofluorescence analysis of GFP-STATc nuclear translocation in GFP-STATc expressing Ax2, pyk3^−^, phg2^−^, and pyk3^−^/phg2^−^ cells.** (A) Ax2/GFP-STATc, (B) pyk3^−^/GFP-STATc, (C) phg2^−^/GFP-STATc, (D) pyk3^−^/phg2^−^/GFP-STATc. Log phase cells were washed twice with Soerensen buffer, transferred to coverslips and allowed to settle for 15 minutes. After treatment with 100 mM sorbitol for 0, 2, 3, 4, 5 and 8 minutes, cells were fixed with methanol and the nuclear translocation of GFP-STATc was analysed with a fluorescence microscope. Top: Exemplary images. Scale bar: 10 µm. Bottom: Quantification of GFP-STATc nuclear translocation in Ax2 wild-type and mutant cells. For each time point, we analysed 150 cells per experiment. The number of cells showing either clear cytosolic (black bar) beginning nuclear (grey bar) or prominent nuclear (white bar) localization of GFP-STATc was determined and the percentage of cells in each of these three categories was calculated. The error bars depict standard deviations of two independent experiments.
(PDF)

Figure S6 **Overexpression of Phg2 causes a strong increase in STATc protein levels.** Ax2 wild-type and Ax2/Myc-Phg2 expressing cells were either treated with 200 mM sorbitol for 15 min or left untreated and total cell lysates were prepared. Proteins were separated by SDS-PAGE and transferred to nitrocellulose. Western blot analysis was performed with antibodies specific for total (7H3) and tyrosine phosphorylated STATc (CP22), for Myc-Phg2 (mAb 9E10), and for endogenous Phg2 (polyclonal Phg2 antibody). Actin was used as loading control and detected with an actin-specific antibody (mAb Act 1–7).
(PDF)
