# Supplementary figures and images for: Identification of the Protein Kinases Pyk3 and Phg2 as Regulators of the STATc-Mediated Response to Hyperosmolarity
Source: PLoS One. 2014 Feb 25;9(2):e90025. doi: 10.1371/journal.pone.0090025 (PMC3934975; doi:10.1371/journal.pone.0090025)

Fig. S1

A

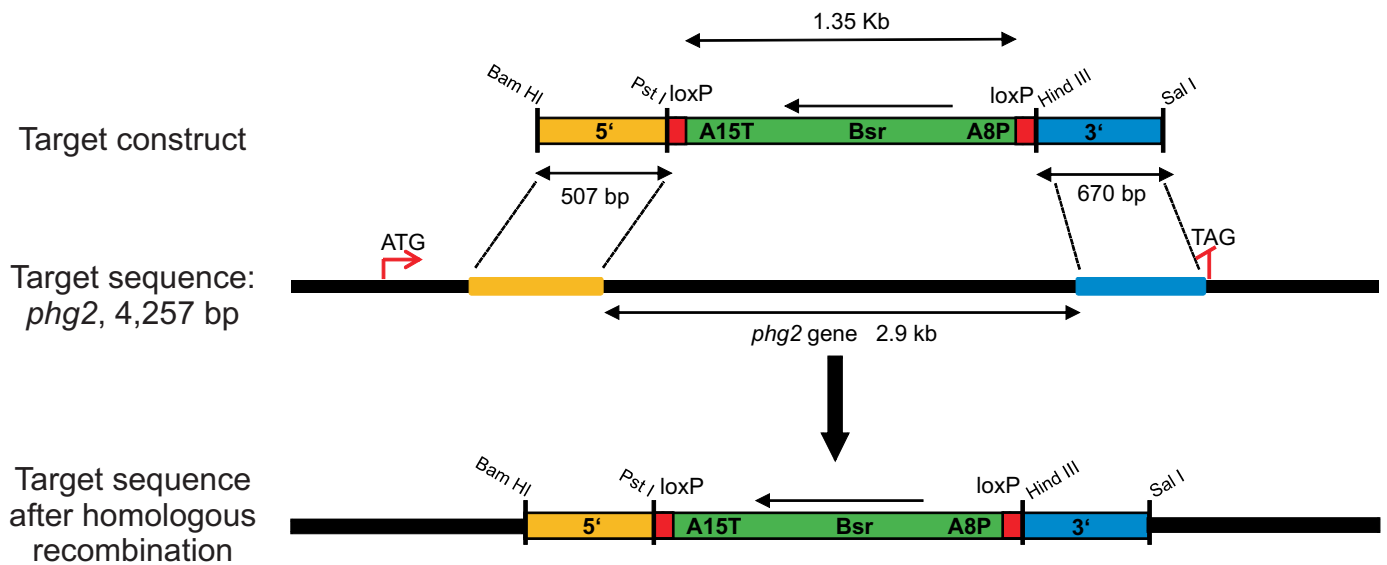

B

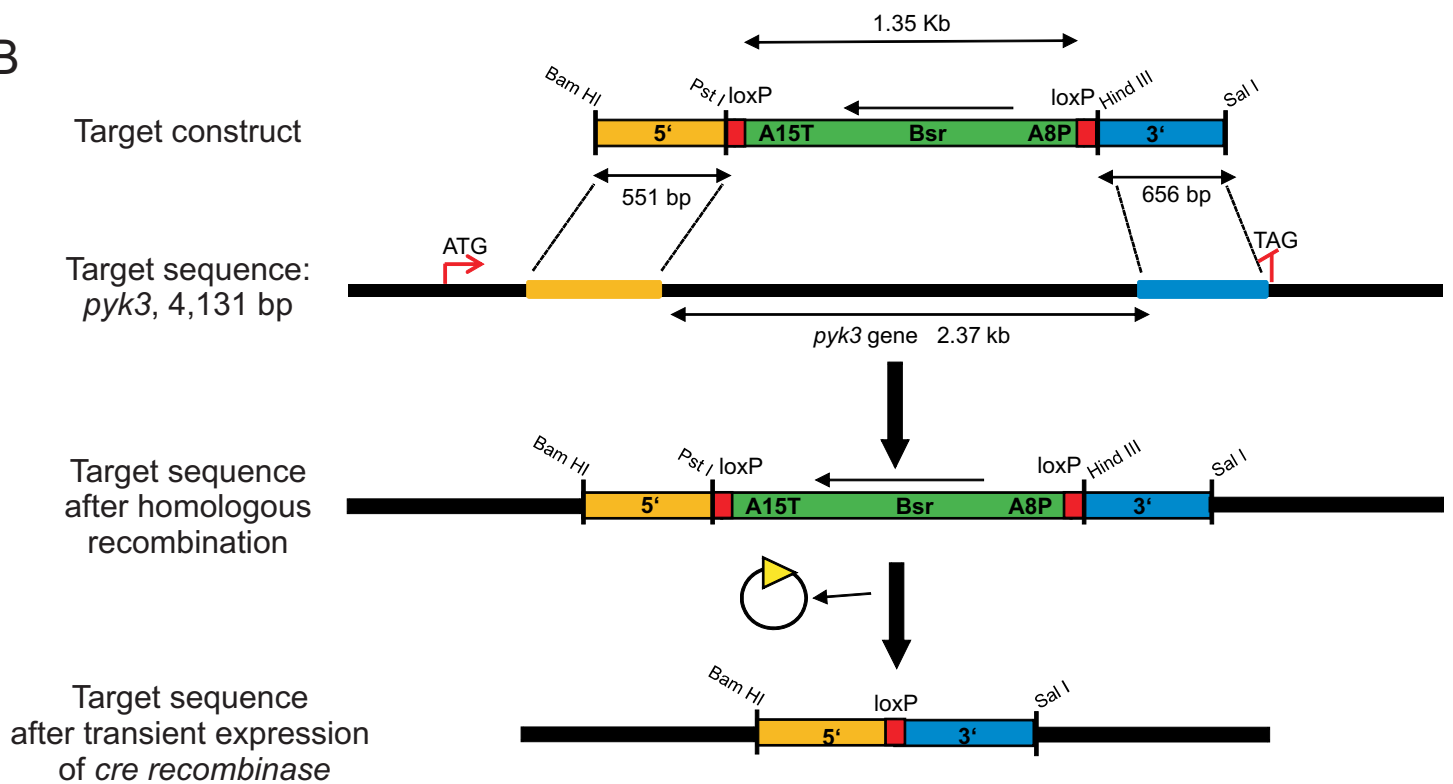

Supplement: Figure S1 — Generation of knock-out mutants. (A) The phg2− strain was generated by replacement of a 2.9 kbp genomic fragment of the phg2 gene that contained part of the tyrosine kinase domain coding sequence with the targeting construct containing the blasticidin resistance cassette flanked by loxP recombination sites. The targeting construct with the blasticidin resistance cassette is shown on top, the genomic sequence of phg2 before the recombination event in the middle, and after the recombination event at the bottom. BamHI, PstI, HindIII, SalI: DNA restriction enzyme sites that were used for the generation of the targeting construct. A8P: Actin 8 promoter. A15T: Actin 15 terminator. Bsr: blasticidin resistance gene. The illustration does not reflect the true scale of actual parts of the disruption vector. (B) The pyk3− strain was generated by replacement of a 2.37 kbp genomic fragment of the pyk3 gene with the targeting strategy as described for phg2−. The pyk3−/phg2− double knock-out strain was generated by transforming the pyk3− strain after transient expression of the cre recombinase (restored blasticidin sensitivity) with the targeting construct for phg2 (shown at the bottom). (PDF) [file pone.0090025.s001.pdf]

Fig. S2

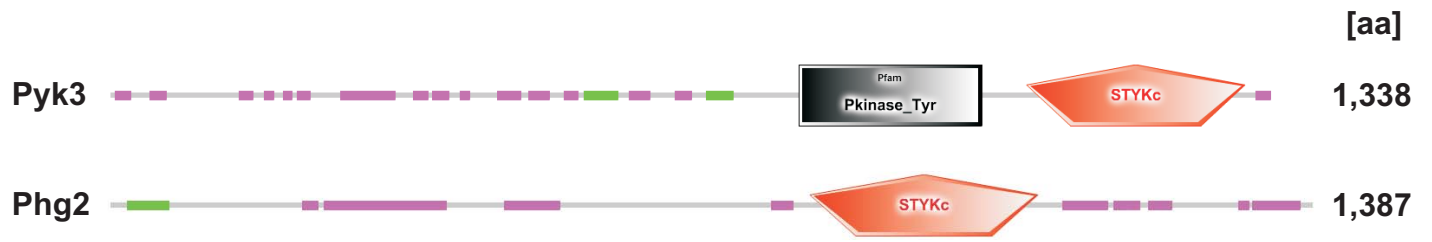

Supplement: Figure S2 — Domain structure of D. discoideum Pyk3 and Phg2. The protein sequences of Pyk3 (DDB_G0289001) and Phg2 (DDB_G0283699) were analysed with SMART (http://smart.embl-heidelberg.de/) to elucidate their domain architecture. STYKc: possible dual-specificity Ser/Thr/Tyr protein kinase domain; green rectangles: coiled coil regions; lilac rectangles: segments of low compositional complexity; aa: amino acids. (PDF) [file pone.0090025.s002.pdf]

Fig. S3

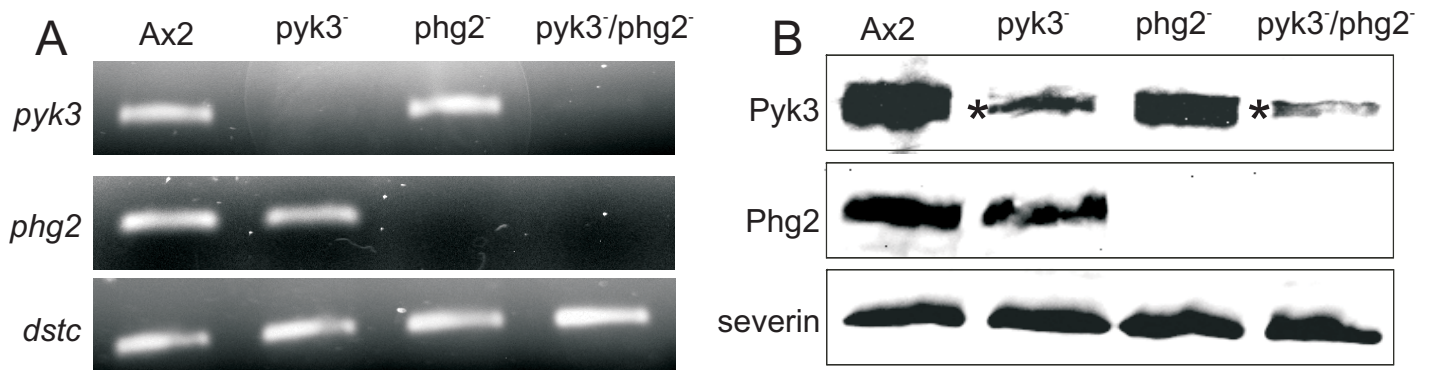

Supplement: Figure S3 — Analysis of the generated pyk3−, phg2− and pyk3−/phg2− knock-out strains. (A) Reverse transcription PCR using primer pairs that amplify small fragments of the 3′-end of the pyk3, phg2, and dstc cDNA, respectively. dstc was used as a positive control. (B) Western blot analysis of total cell lysates of Ax2 wild-type, pyk3−, phg2−, and pyk3−/phg2− cells. Proteins were separated by SDS-PAGE, transferred to nitrocellulose and Western blot analyses with polyclonal pyk3 and phg2 antibodies were performed. Severin was used as loading control and detected with a monoclonal severin antibody (mAb 101-460-2). Please note that the weak band in the pyk3− strain (*) is due to cross-reaction of the Pyk3 antibody. A similar cross-reaction with this antibody was also seen in the independently generated pyk3− mutant in the lab of J.G. Williams (data not shown). (PDF) [file pone.0090025.s003.pdf]

Fig. S4

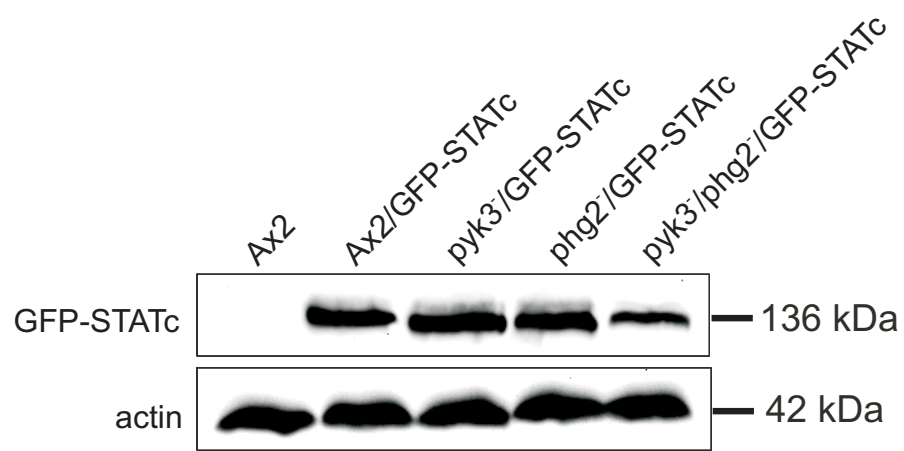

Supplement: Figure S4 — Western Blot analysis of GFP-STATc expression in Ax2, pyk3−, phg2−, and pyk3−/phg2− cells. The expression of GFP-STATc was monitored by Western blot analysis of total cell lysates with a monoclonal GFP antibody (mAb K3-184-2). Actin was used as loading control and detected with a monoclonal actin antibody (mAb Act1-7). (PDF) [file pone.0090025.s004.pdf]

Fig. S5

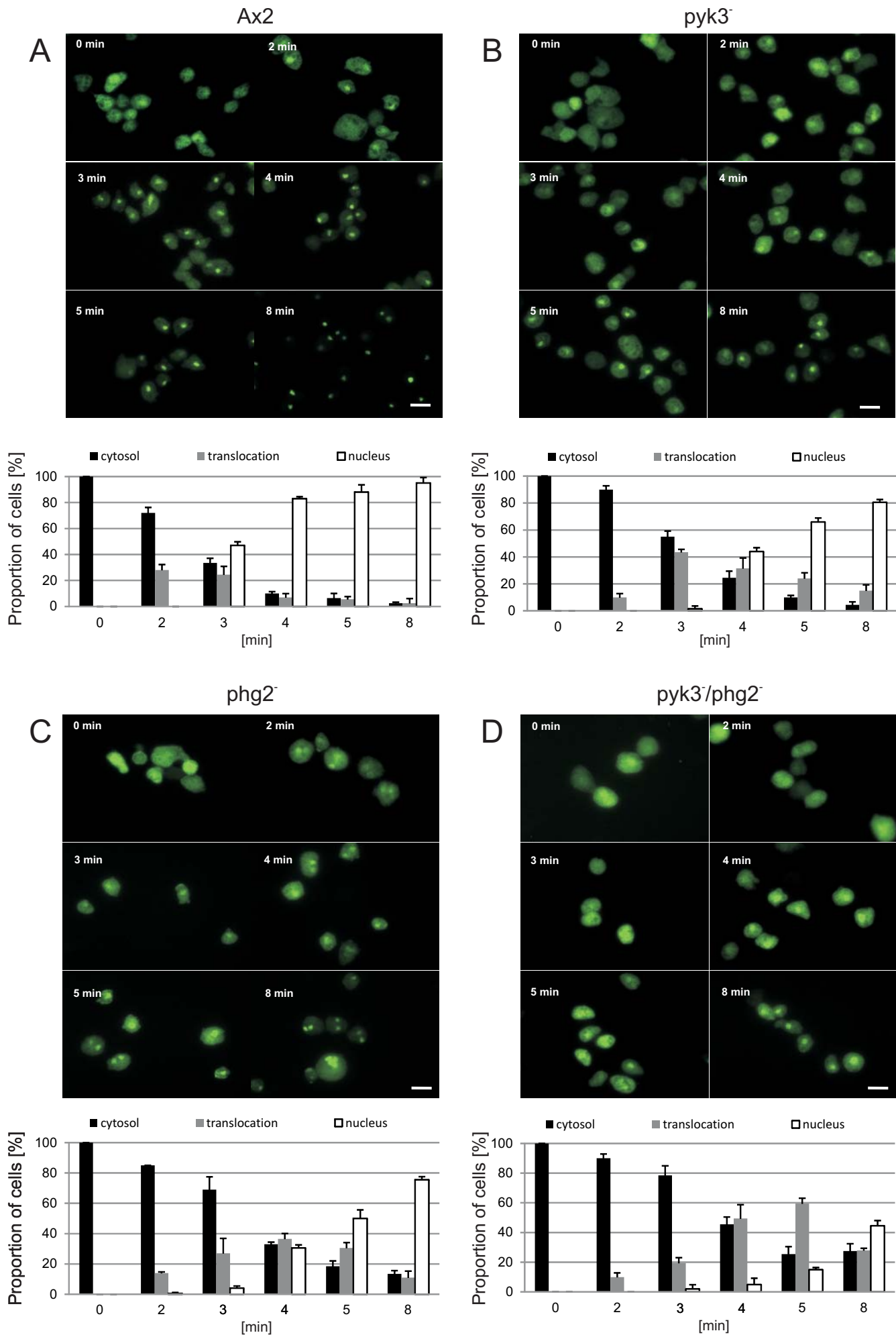

Supplement: Figure S5 — Immunofluorescence analysis of GFP-STATc nuclear translocation in GFP-STATc expressing Ax2, pyk3−, phg2−, and pyk3−/phg2− cells. (A) Ax2/GFP-STATc, (B) pyk3−/GFP-STATc, (C) phg2−/GFP-STATc, (D) pyk3−/phg2−/GFP-STATc. Log phase cells were washed twice with Soerensen buffer, transferred to coverslips and allowed to settle for 15 minutes. After treatment with 100 mM sorbitol for 0, 2, 3, 4, 5 and 8 minutes, cells were fixed with methanol and the nuclear translocation of GFP-STATc was analysed with a fluorescence microscope. Top: Exemplary images. Scale bar: 10 µm. Bottom: Quantification of GFP-STATc nuclear translocation in Ax2 wild-type and mutant cells. For each time point, we analysed 150 cells per experiment. The number of cells showing either clear cytosolic (black bar) beginning nuclear (grey bar) or prominent nuclear (white bar) localization of GFP-STATc was determined and the percentage of cells in each of these three categories was calculated. The error bars depict standard deviations of two independent experiments. (PDF) [file pone.0090025.s005.pdf]

Fig. S6

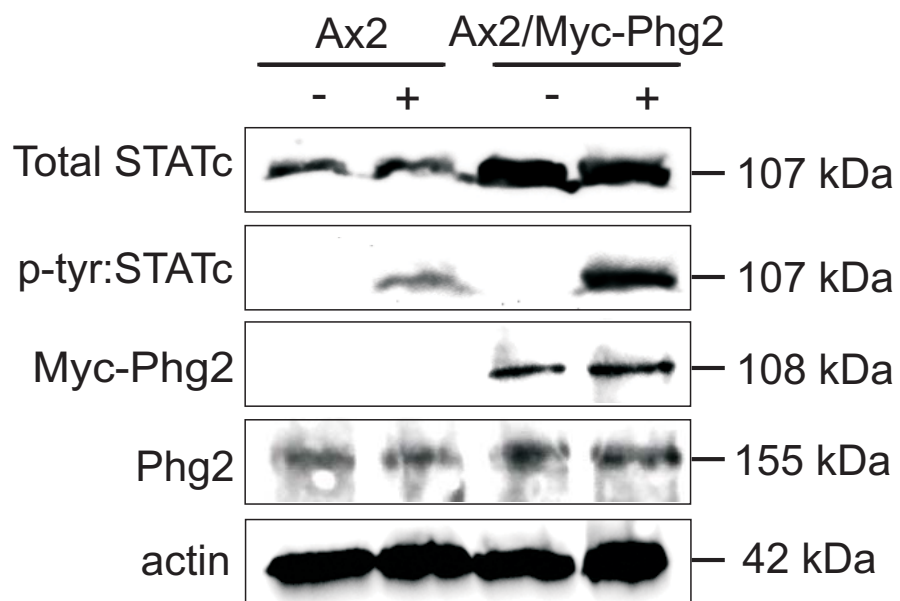

Supplement: Figure S6 — Overexpression of Phg2 causes a strong increase in STATc protein levels. Ax2 wild-type and Ax2/Myc-Phg2 expressing cells were either treated with 200 mM sorbitol for 15 min or left untreated and total cell lysates were prepared. Proteins were separated by SDS-PAGE and transferred to nitrocellulose. Western blot analysis was performed with antibodies specific for total (7H3) and tyrosine phosphorylated STATc (CP22), for Myc-Phg2 (mAb 9E10), and for endogenous Phg2 (polyclonal Phg2 antibody). Actin was used as loading control and detected with an actin-specific antibody (mAb Act 1–7). (PDF) [file pone.0090025.s006.pdf]
